# 

**Published:** 1907-11

**Authors:** 


Sixty-third Year.

VOL. LXIII. NOVEMBER, 1907. No. 4

### BUFFALO MEDICAL JOURNAL

Established 1845 by AUSTIN FLINT, M. D.

LIBRARY Indexed S. G. O.

LIBRARY SURGEON GENERAL'S OFFICE NOV 4-1907

EDITOR:

WILLIAM WARREN POTTER, M. D.

ASSISTANT EDITORS:

WILLIAM C. KRAUSS, M. D. NELSON W. WILSON, M. D.

ASSOCIATE EDITORS:
JAMES WRIGHT PUTNAM, M. D. ERNEST WENDE, M. D.
JOHN PARMENTER, M. D. JOHN A. MILLER, PH. D.
MAUD J. FRYE, M. D.

